# Exploring Student Perspectives and Experiences of Online Opportunities for Virtual Care Skills Development: Sequential Explanatory Mixed Methods Study

**DOI:** 10.2196/53777

**Published:** 2024-08-21

**Authors:** Lorelli Nowell, Sara Dolan, Sonja Johnston, Michele Jacobsen, Diane Lorenzetti, Elizabeth Oddone Paolucci

**Affiliations:** 1 Faculty of Nursing University of Calgary Calgary, AB Canada; 2 Werklund School of Education University of Calgary Calgary, AB Canada; 3 Cumming School of Medicine University of Calgary Calgary, AB Canada

**Keywords:** virtual care, online teaching and learning, mixed methods research, virtual care, development, mixed method study, online, care, student, students, online learning, virtual learning, interview, experience, educational, technology, nursing, medicine, allied health, teaching

## Abstract

**Background:**

Caring profession students require skills and competencies to proficiently use information technologies for providing high-quality and effective care. However, there is a gap in exploring the perceptions and experiences of students in developing virtual care skills within online environments.

**Objective:**

This study aims to better understand caring professional students’ online learning experiences with developing virtual care skills and competencies.

**Methods:**

A sequential explanatory mixed methods approach, integrating both a cross-sectional survey and individual interviews, was used to better understand caring professional students’ online learning experiences with developing virtual care skills and competencies.

**Results:**

A total of 93 survey and 9 interview participants were drawn from various faculties, including students from education, nursing, medicine, and allied health. These participants identified the barriers, facilitators, principles, and skills related to learning about and delivering virtual care, including teaching methods and educational technologies.

**Conclusions:**

This study contributes to the growing body of educational research on virtual care skills by offering student insights and suggestions for improved teaching and learning strategies in caring professions’ programs.

## Introduction

### Background

The COVID-19 pandemic created a prime opportunity for higher-education institutions to reevaluate how online education is provided. More specifically, the pandemic considerably changed the ways in which education and care are provided across the caring professionals including teachers, physicians, nurses, and allied health professionals. It has become essential to ensure that caring professionals have the required skills and competencies to adapt and navigate this new virtual care landscape. The pandemic also uncovered a range of virtual care skills and competencies that caring professions students will need to thrive, reflecting a new era where remote care is often facilitated by information technologies to facilitate high-quality and effective care.

Traditionally, caring profession students have learned the required skills and competencies of their profession through in-person lectures, in-class group experiences, and work-integrated placements with educators and professionals (eg, kindergarten to 12th-grade classrooms, hospital settings, and counseling centers) [1,2]. The COVID-19 pandemic accelerated a shift to virtual learning and caring contexts, where students both learned about their profession and cared for the public, using information technologies for effective training [3-5].

Virtual care is care provided through digital online collaboration platforms where caring professionals use interpersonal and technological skills to provide care to clients in a virtual setting, including a variety of activities such as telemedicine, distance consultation, remote counseling, and online education. The authors conducted a recent systematic review, which identified that students value the benefits of and are eager to learn how to use digital technologies in preparation for their future roles in caring professional practice [6]. The review highlighted that student learning was best supported when online learning activities and technology aligned and closely replicated real-world situations [6]. Furthermore, students who engaged in online learning about virtual care were more likely to incorporate virtual care into their future professional practice [6].

### Objectives

Despite these findings, there remains a need to identify the teaching and learning conditions that may optimize e-learning experiences to support caring profession students in developing virtual care skills and competencies [6]. More research is needed to explore the perceptions and experiences of students in developing virtual care skills within online environments. We therefore conducted a study to answer the following research questions:

1. How do caring professions students *describe* the components of current online teaching and learning innovations that support their development of virtual care skills and competencies?
2. What are caring professions students’ *experiences and perceptions* of online learning opportunities for developing their virtual care skills and competencies?
3. What are the *facilitators and barriers* to creating and engaging in online learning opportunities to support student’s development of virtual care skills and competencies?

## Methods

### Design

We used a sequential explanatory mixed methods approach for data collection, analysis, and integration of quantitative and qualitative data [7,8]. We used a cross-sectional survey and individual interviews to understand caring professional students’ online learning experiences with developing virtual care skills and competencies. Integration between the 2 phases occurred when the survey responses were used to identify potential participants for follow-up interviews. This process involved analyzing the survey data to purposively sample interview participants that represented diverse perspectives and characteristics of interest. Subsequently, the qualitative interviews provided a deeper, context-rich understanding of the survey findings, allowing for a more comprehensive exploration.

### Sample and Participants

Voluntary participation was sought from students in caring professions, encompassing education, medicine, nursing, and allied health, within a midsized research-intensive institution located in western Canada.

### Data Collection

We developed an online survey following well-established methods [9] to examine components of current e-learning opportunities, explore students’ perceptions and experiences of these opportunities, and identify the facilitators and barriers to engaging in online learning that support the development of virtual care skills and competencies. The survey was informed by a systemic review previously completed by the authors [6]. To our knowledge, no previously validated tools have been published that could be used to answer our research questions. To ensure measurement-related validity (face and content), the survey was piloted with 10 caring profession students who did not subsequently participate in the formal study. Their suggested edits were incorporated before survey distribution [7,10,11]. Survey items were a mix of Likert, closed-ended, and open-ended questions including demographic questions and questions about educational experience, instructional methods, satisfaction and effectiveness of technologies and educational methods, and preparedness to provide virtual care. Surveys were distributed securely online using Qualtrics (Qualtrics International Inc), and students were recruited via university email, Twitter, Instagram, and Facebook [12,13]. Due to individual faculty policies, only students from education, nursing, and medicine were sent a recruitment email directly. Social media recruitment targeted students from the caring profession faculties of education, medicine, nursing, and allied health within this western Canadian university (approximately 8000 students). Informed consent was assumed if the survey was completed. All survey participants were also asked to provide an email address if they were interested in participating in a follow-up interview. Interviews were conducted to better understand students’ perceptions and experiences vis-à-vis the research questions.

A semistructured interview guide was developed based on the findings from the authors’ published systematic review and online survey responses. While no theoretical frameworks were used in the creation of the interview guide, the categories, domains, and concepts identified in the systematic review helped inform the interview questions. All survey participants who provided their email were contacted to participate in a 30- to 60-minute interview conducted via Zoom (Zoom Video Communications). Before each interview, oral consent was obtained and documented. The interviews were audio recorded and transcribed verbatim.

### Data Analysis

Closed-ended online survey responses were downloaded from Qualtrics and imported into the SPSS (version 28; IBM Corp). The characteristics of the study population (eg, age, gender, faculty, and experience) were analyzed using descriptive statistics. Differences in the distribution across the data set were summarized and displayed in tabular and graphical formats [11]. In addition, univariate analyses were conducted on continuous data where appropriate [11]. Several 1-way ANOVA tests were completed to assess potential differences in satisfaction and preparedness scores based on various demographic factors, including gender, age, faculty, or experience with online teaching and learning technologies. To analyze the relationships with age, participant ages were grouped into 5 categories: ≤19, 20 to 29, 30 to 39, 40 to 49, and ≥50 years, making the data more readable and more amenable to post hoc analysis should significance be found.

All open-ended online survey responses and interview transcripts were assigned unique identifiers and imported into NVivo software (version 14; QSR International) for qualitative data management. Qualitative data were analyzed thematically via induction to transform data from individual sources to common themes [14,15]. To familiarize ourselves with the data, the entire qualitative data set was initially read independently by 2 researchers (LN and SD) who met regularly to discuss, develop, and ensure mutual understanding and agreement of the initial codes. Detailed categorical code descriptions were created using NVivo software. Each data set was independently examined within NVivo by the same 2 researchers who coded sections of text, and the independent data files were merged to assess alignment and divergence. Any divergence between the researchers was discussed and resolved without the use of a third party. Larger team meetings were held with all authors to examine and further refine patterns in the data and confirm themes and subthemes. Written memos and meeting minutes were used to record our data analysis process. We followed the Standards for Reporting Qualitative Research (SRQR) in our research and reporting processes [16].

### Ethical Considerations

We were granted permission to conduct this research through the University of Calgary's Conjoint Health Research Ethics Board (REB22-1054). Students were invited to participate voluntarily, with the assurance that their involvement in the survey would be anonymous and would not impact their standing at the university. Completion of the survey was considered as implied consent. For the interviews, all participants provided informed verbal consent before engaging in an interview. These interviews were conducted by a graduate student who did not know and was not responsible for teaching the participants. The audio recordings were professionally transcribed and then anonymized by the graduate student before sharing any data with the larger research team. To further maintain participant anonymity, unique identifiers were assigned to each participant, and the data were aggregated accordingly.

### Rigor

Several techniques were used to ensure rigor in our study. Regular team meetings created occasions for debriefing, reflexivity, and purposeful questioning of our interpretations [17]. A detailed audit trail including codebooks, meeting minutes, and shared files was maintained to document all study decisions [18]. While 2 researchers coded all qualitative data, all decisions about themes and subthemes were assessed by the larger research team. We returned to the raw survey and interview data to further verify our findings and ensure that our findings adequately displayed the student participants’ voices [17].

## Results

### Participant Demographics

A total of 107 students started the survey, and 93 (86.9%) completed the survey. Of the 93 survey participants, 15 (16%) agreed to be contacted for a follow-up interview, of which 9 (10%) responded and completed an interview. Table 1 presents the participant demographics for the survey and interviews.

**Table 1:** Participant demographics.

Demographic subcategory			Survey participants (n=93), n (%)		Interview participants (n=9), n (%)
**Age group (y)**					
	<20	11 (12)		0 (0)	
	20-29	39 (50)		2 (22)	
	30-39	22 (24)		2 (22)	
	40-49	14 (15)		2 (22)	
	≥50	5 (5)		3 (33)	
	Not disclosed	2 (2)		0 (0)	
**Gender**					
	Man	15 (16)		2 (22)	
	Woman	74 (80)		7 (78)	
	Gender diverse^a^	4 (4)		0 (0)	
**Faculty**					
	Education	22 (24)		2 (22)	
	Medicine	27 (29)		0 (0)	
	Nursing	42 (45)		7 (78)	
	Allied health	2 (2)		0 (0)	
**Experience (self-report)**					
	Beginner	13 (14)		1 (11)	
	Intermediate	33 (35)		2 (22)	
	Expert	47 (51)		6 (67)	

^a^Gender diverse includes gender fluid, nonbinary, queer, and individuals who prefer not to disclose due to the need for maintaining anonymity, especially with small numbers.

### Quantitative Results

Student survey respondents (n=89) reported that the faculty used a variety of online instructional methods and various teaching and caring technologies to help develop students’ virtual care skills. For instance, the most-reported instructional technology that respondents (n=93) reported being exposed to included audio conferencing and videoconferencing (n=50, 54%), online learning systems (n=50, 54%), and online media (n=35, 38%). Other instructional technology included simulated health records (n=25, 28%) and telehealth monitoring systems (n=5, 1%). Similarly, the most frequently reported instructional methods included online modules (n=77, 83%), reflection on learning (n=66, 71%), and online discussion boards (n=65, 70%). Other instructional methods included consultation with clients (n=25, 27%) and demonstration of remote care (n=8, 9%).

Students were asked how satisfied they were with the online teaching and learning strategies that were used to help them develop virtual care skills. Of the students who responded to the question (n=89), only 9 (10%) indicated that they were not satisfied, with others indicating they were satisfied (n=20, 23%) or somewhat satisfied (n=60, 67%). Students were also asked how prepared they felt to use virtual care skills in their future practice. Of the students who responded to the question (n=64), 11 (17%) indicated they were not prepared, with other indicating they were prepared (n=22, 25%) or somewhat prepared (n=52, 58%). The ANOVA test results did not reveal any statistically significant differences among the groups (gender, age, faculty, and experience) for both satisfaction and preparedness scores. The full ANOVA test results and exact *P* values are presented in Table 2.

**Table 2:** ANOVA results.

Scores and groups		*F* test (*df*)	Mean square	*P* value
**Satisfaction**				
	Gender	1.514 (2)	0.470	.23
	Faculty	1.806 (3)	.552	.15
	Experience	0.106 (2)	.034	.90
	Age	1.353 (4)	.396	.26
**Preparedness**				
	Gender	0.026 (2)	.011	.98
	Faculty	1.642 (3)	.673	.19
	Experience	0.144 (2)	.063	.87
	Age	1.380 (4)	.569	.25

### Qualitative Findings

#### Overview

**Figure 1:**
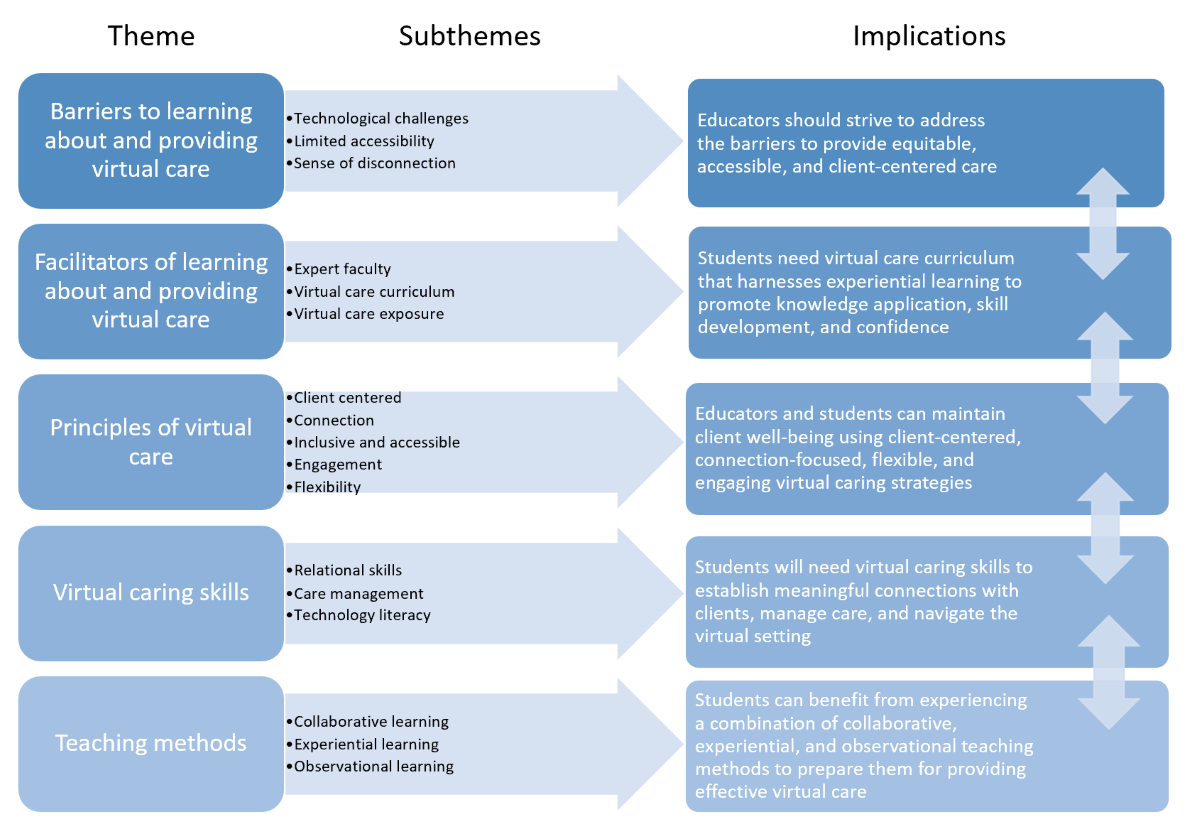
Overview of themes, subthemes, and implications.

Figure 1 provides a high-level overview of the 5 themes and their corresponding 16 subthemes derived from the qualitative data, along with potential implications. These findings are explored in further depth in the sections that follow. Overall, interview participants lacked exposure to a robust virtual care curriculum. These findings represent their perspectives on the limited exposure that they did have and perspectives on what could be valuable to students in the future. Participants’ limited exposure to virtual care learning varied as participants were recruited from different faculties and programs. Students reflected on how learning in an online setting had helped them to gain or enhance virtual care skills.

#### Barriers to Learning About and Providing Virtual Care

Students identified several barriers to online learning related to developing virtual care skills for their future practice: technological challenges, limited accessibility, and a sense of disconnection.

##### Technological Challenges

Interviewees identified several key challenges to their active participation in online learning aimed at fostering virtual care skills and competencies. These challenges included streaming problems, difficulties navigating new technologies, and the unreliability of the technology. Students expressed their struggles with web-based learning platforms, which can often be unpredictable and unreliable:

The system would kick the whole class out and people would have to log back in again if too many people tried to turn their cameras on. Even in cases where people were trying to fight against that black screen, sometimes the technology would fail and then you’d be stuck in the same situation.

P6, interview, education student

Technology challenges further impacted the students’ ability to provide virtual care to clients. Students commented that in addition to learning how to use the technology themselves, they needed to guide clients using unfamiliar technology. In addition, they needed to accommodate technology failures when they occurred:

Navigating or teaching clients how to navigate which platforms to use... some clients aren’t well-versed in using this platform, I think that’s definitely a barrier.

P1, interview, nursing student

Students commented on the time implications when technology failed to work:

One of the things that we use here in my community is telehealth, but even that can be terribly complicated... you get together and then the technology does not work and then everybody has to plan again.

P3, interview, nursing student

Challenges with technology clearly impacted students’ learning as well as their ability to provide excellent and timely care to their clients.

##### Limited Accessibility

Issues with accessibility, particularly inadequate internet connections, hindered students from engaging in learning opportunities that are crucial for developing essential virtual care skills. One student stated as follows:

If my wifi cut out, I would completely miss what somebody said. And the bigger of a group you have, the more technological barriers we faced with maybe not the best sound quality or somebody’s camera maybe not working.

P8, interview, nursing student

One student discussed the challenge of providing virtual care to a population that might lack access to the technology used by providers. The student emphasized the necessity of being adaptable and responsive to the diverse needs of clients:

For example, text messaging can be a good thing. It’s shown in the literature and different studies that being able to stay connected with a client through text messaging that’s been set up that you’re going to text them twice a week as a check-in, for example, was one of the things that came up. Well, that’s great if, of course, your client has a cell phone, or has reliable access. So, then you look at what are the client strengths? Do they have access to a cell phone? If they don’t, well then that’s not going to work, so you’re going to need to come up with something else.

P3, interview, nursing student

Inadequate access to mobile devices and robust internet connections can impact student learning and the types of care they can provide to clients.

##### Sense of Disconnection

Students were worried that providing care in a virtual environment could restrict their ability to develop human connections with clients or provide specific therapeutic services that require in-person interaction. For example, therapeutic touch is an important caring skill used in face-to-face interactions:

Because in person you can make physical contact, obviously therapeutic and appropriate with a person, versus online. I can’t really hug my computer. It’s more difficult to display that physical human connection. I mean, sure, you can express empathy through your words and your intent, but that’s a little bit different than that face-to-face value. So, I could see that as another barrier.

P8, interview, nursing student

Students who were learning to both provide care and teach in online settings spoke of the lack of personal connection and the challenge of establishing a sense of community with their clients as significant barriers to the provision of care:

...[N]obody turned their camera on. It just felt like I was lecturing to my computer screen alone in a room... it wasn’t a strong community... I tried to get them to turn their cameras on, but I just couldn’t break that wall down.

P6, interview, education student

Another student spoke about the complexity of providing appropriate personalized care over the phone, noting they were unable to connect with clients by reading their body language:

I do work at a crisis center where I am over the phone, and we don’t have that face-to-face interaction. And then I work in the emergency department in mental health. And so, a lot of the body language, the information you can collect when you have a patient in person is very different than the information that you need to collect over the phone.

P9, interview, nursing student

The importance of creating meaningful connections with clients was confirmed by other student respondents who noted that “being online detracts from the real human experience” (P83, survey, medical student)” and confirmed the challenges associated with “convey[ing] compassion online and… develop[ing] relationships with large online groups” (P8, survey, nursing student).”

#### Facilitators of Learning About and Providing Virtual Care

Students identified several facilitators of online learning related to developing virtual care skills for future practice, including expert faculty, virtual care curriculum, and virtual care exposure.

##### Expert Faculty

Expert faculty are needed to facilitate the learning of virtual care skills. While students indicated that the faculty should be skilled in educating in online environments to provide a smooth, engaging atmosphere for students, they also reported that their experience varied by the educator:

Some were excellent at facilitating ways to participate online and others didn’t have the skills needed to build an online community of learners.

P44, survey, education student

Students also noted that it is important for faculty to be “competent in using online technology and be held accountable to upload information and provide feedback in a timely manner being available to students as needed” (P56, survey, nursing student). Students discussed the potential benefit of having the faculty who have experience in providing virtual care and the opportunity for faculty members to reach out to other departments for expert guidance regarding teaching virtual skills in online environments, with 1 student stating as follows:

...[T]hat mentorship component is when you are following... a mentor who’s in that field who can help you relate to the material that you’re learning, I find that to be really helpful, the interactive component of it.

P9, interview, nursing student

As noted by student participants, expert faculty can also role model and encourage online etiquette, foster psychological safety in the classroom, and ensure that there are interactive activities to facilitate a shared experience.

##### Virtual Care Curriculum

Students indicated a genuine desire for a virtual care curriculum; however, none of the interviewed participants had received robust virtual care training in their programs of study, such as a virtual care course. Some participants indicated that the topic would be brought up as a discussion around different ways that professionals may interact with clients rather than a devoted topic. Participants thought it would be beneficial if their professional educational programs provided a virtual care course or threaded a virtual care curriculum to address topics such as relevant technology, information on remote care equipment (eg, telemonitoring devices), rapport building in the virtual setting, and limitations of virtual care. Students urged that virtual care is a reality that needs to be addressed in professional educational programs that teach caring skills. Students in this study expressed a clear desire to learn how to teach remotely. One student remarked as follows:

Oh, definitely have a remote teaching course... how to navigate Zoom as an instructor or in Google Classroom... how to navigate some of the online assessments... That would be awesome. I would totally take that course

P4, interview, education student

As another student confirmed as follows:

[W]e focus so much on the bedside nursing, on the clinician approach to care, that we forget about the tremendous opportunities that come about with remote care as well as the need to have healthcare workers in the remote care setting.

P8, interview, nursing student

##### Virtual Care Exposure

Students conveyed the desire to be exposed to virtual care during their educational programs by shadowing a virtual care provider, practicing virtual care skills with clients, viewing online virtual care demonstrations, or role-playing and simulating virtual care environments where they could demonstrate their virtual care competencies in a psychologically safe environment. Exposure to individuals working in remote health care settings was viewed as highly beneficial and most effective when students understand the diverse scope of practice:

I feel like having more individuals who work in the remote setting and being able to shadow them or have a clinical rotation in a remote setting would definitely help if it was more inclusive of the broad scope of practice of healthcare practitioners.

P8, interview, nursing student

Survey respondents provided additional statements suggesting specific educational strategies that could be implemented to enhance learning in online settings, such as “some virtual simulations would be a great addition to the tools already used” (P61, survey, nursing student), “Exposure and experience with various technologies, and careful consideration of the intended goals and benefits/detriments of using these technologies” (P9, survey, education student), and “I wish educators focused more on demonstrations and explaining how this will work in ‘the real world’” (P10, survey, nursing student).” According to the students, the more exposure to virtual care, the better, and thoughtfully integrating practical experiences with technology can support more effective online learning.

#### Principles of Virtual Care

We identified 5 principles of virtual care: client centered, connection, inclusive and accessible, engagement, and flexibility. These principles reflect what students reported as important factors in their ability to learn about and demonstrate caring skills in a virtual environment.

##### Client Centered

Students recognized the value of client-centered learning for their transition to caring practice. Students expressed that when learning in an online environment, they wanted to feel cared for and that their well-being was a priority; this translated to experiences they wanted to create for their current and future virtual clients. Students expressed how the client-centered approach involves individually assessing client needs and adapting to effectively meet them within the online environment:

Understanding how we can adapt and meet client needs better just in the environment alone I think is something else that I kind of have taken away from that experience.

P1, interview, nursing student

Online learning throughout the pandemic also helped students recognize that people are complex and more than a name or diagnosis:

I think the pandemic and online learning pulled back that veil a bit and showed how complex people are. I hope that an awareness of that complexity and the needs that come with being whole people... carries forward in teaching both online and in person.

P6, interview, education student

One student further identified how being client centered could also inform the questions that they may ask and the care they may provide for future remote clients:

Not just seeing a person as a diagnosis but seeing them as so much more. Seeing is how their community, the people in their lives affect their quality of life and wellbeing, as well as the resources they have access to or don’t have access to, and making meaningful changes to them in a remote setting.

P8, interview, nursing student

The pandemic raised a newfound awareness of human complexity and integrating a more holistic approach to both online and in-person teaching was viewed as valuable by students. Students emphasized the importance of seeing the “human” as a dynamic member of a larger system. Hence, caring for people necessitates recognizing individual and systemic influences of care.

##### Connection

Students expressed the importance of connection during their online learning. They emphasized the value of relationship building and discussed how a lack of connection can adversely impact engagement. They appreciated the faculty who invested time in checking in with them and became acquainted with them as individuals. They viewed this ability to connect as an important skill to develop in themselves. Other students noted that taking the time to build community was essential for building connections with both colleagues and clients:

Online learning really showed me the value of slowing down a little bit. While still getting through all the content you need to get through in a good way, it’s okay to also have moments... that are just about community building... and checking in with people.

P6, interview, education student

They expressed that relationships were built through shared experiences:

I have found that relationship building is key... these relationships are what stimulate not only engagement with the material but also motivation to participate and apply what one is learning.

P15, survey, nursing student

Students prioritized connection as part of their virtual learning and virtual care practices.

##### Inclusive and Accessible

Students remarked on the extent to which educational technology has made learning and caring more inclusive and accessible. Students noted how educational technology has created new ways of engagement, particularly benefiting those who may have previously been excluded:

Online and virtual open up opportunities to learn and interact in multiple ways such as text alongside audio. This has meant people with disabilities could join and participate more.

P67, survey, medical student

It has also allowed those students who live outside of urban centers or who are balancing various life priorities to learn in new ways:

It has made it so I as a working mother of 2 children can continue my education without feeling I must put these other things in my life on hold. Online teaching has meant I get to pursue my career aspirations sooner than I ever expected. It is very exciting for me.

P15, survey, nursing student

Students also reflected on how virtual care has provided a means for clients outside of large urban areas to access care that they may not have been able to previously:

It tends to be a lot more accessible for clients... we can adapt and meet client needs better just in the environment alone I think is something else that I kind of have taken away from that experience.

P1, interview, nursing student

Students recognized and appreciated the transformative impact of educational technology, particularly online and virtual platforms; this technology has made learning and caring more inclusive, accessible, and equitable, benefiting learners with diverse life commitments and clients residing in rural locations.

##### Engagement

Students conveyed the belief that engagement is a key principle in online learning of virtual care skills. Students expressed that engagement could be achieved in many ways including engaging in in-class activities and group work, having breakout rooms, leaving time for student questions, sharing experiences, role-playing, and using multimodal content. One student remarked on how they observed an educator modeling an engaging strategy and that they considered how they would use that strategy in their own practice of virtual care:

I learned a lot about different ways... to be present... through the use of my voice and through the use of my face and the video.

P2, interview, nursing student

One student discussed how a professor embraced innovation to enhance engagement in the virtual setting during the challenging early days of the COVID-19 pandemic:

Because I was taking a class online that was land-based learning. That’s a really hard topic to transition to online because you’re off the land in some ways while still being connected to the land in others. The instructor did this really innovative and fascinating thing where they would independently on their own safely go out onto the land and they’d take videos and make little audio recordings of their encounters with the land to show how we can still be land-based even when we’re not together in the same space.

P6, interview, education student

##### Flexibility

Students identified flexibility as an essential principle in learning and applying virtual care skills. Students noted how the inherent nature of online learning provided them with the necessary flexibility to learn content and skills at their own pace and schedule, a skill that can be carried forward to their virtual care practice. Some students noted that the flexibility in online learning allowed them to learn optimally in a way that best suited them:

Online learning means I can learn at my own pace in an environment that is more comfortable and therefore more conducive to learning. Without the time pressure, I could redo things that I struggled with and go faster through other parts.

survey respondent

Students observed flexibility in virtual care when their educators offered flexible communication, flexible office hours, and the freedom to choose assignment topics within the course objectives. This flexibility empowered students to engage more effectively and tailor their learning experience to their unique needs and interests:

There’s more onus placed on the student to learn. What’s beautiful about that, I think, is that, at least from my experience, the learning really was driven by me. There’s a certain topic or there’s a certain parameter around what my learning was about or what I was to achieve, but how I went about doing that, the questions I asked, that really became a personal journey for me rather than fulfilling somebody else’s preset plan for my learning.

P5, interview, nursing student

That same student went on to use that modeled behavior to inform the development of their own virtual care and teaching skills:

I think that there is still similarities there in terms of even just communication, and how is that shaped or modified, or even bettered because of my experience of being a student and my own personal journey with learning. But then be able to communicate better with my students as a result of that rather than a prescript, predetermined pathway. And giving my students that opportunity as well to explore the world according to them, really.

P5, interview, nursing student

Students associated flexibility in access, choice of topics, meeting times, and personalization with optimal online learning and virtual care practice.

#### Virtual Care Skills

Students identified many essential skills needed to provide virtual care. The skills were divided into 3 main categories: relational skills, care management skills, and technology literacy.

##### Relational Skills

Relational skills such as communication, relationship building, ensuring psychological safety, and conveying empathy were viewed as essential for providing care in virtual settings. Students expressed the importance of paying attention to nonverbal cues, voice tone, and active listening when working in virtual contexts:

I think it definitely did help to develop communication techniques over the phone that were mostly verbal, because we lost the aspect of most of the time having non-verbal communication and the ability to build rapport face-to-face. So I think just in terms of dialogue and building rapport over just purely verbal communication, I would say it definitely was beneficial in having more practice to do that.

P1, interview, nursing student

As another student confirmed as follows:

I do find that my skills that I’ve learned and communication skills that I’ve learned doing the online Zoom classes, I’ve been able to utilize some of those skills with delivering remote care to patients...my learning that I’ve done in my program, I’ve been able to put directly to use at work. It’s been very helpful.

P7, interview, nursing student

One student conveyed the significance of small relational moments in building a community during the pandemic:

It actually became about connection and community as well, because I don’t know...I can’t speak for anyone else during the pandemic or in that particular time, but classes, online classes, were really my point of seeing other people outside my immediate bubble when we were all in isolation. Those opportunities to connect and chat in those breakout rooms, both about course content and not about course content, were really nice opportunities at the time.

P6, interview, education student

Students discussed the importance of establishing rapport and building relationships with clients. They remarked on how this was challenging but essential to demonstrating virtual care. One student shared how online learning enhanced the ability to build rapport and relationships with clients:

We talked about the rapport building and that relational aspect to remote care that I think is really important, that I think online learning really hones those skills. It does also help with the assessment skills.

P9, interview, nursing student

Students expressed the significance of cultivating psychological safety when delivering virtual care, as this would allow the client to feel more comfortable and at ease during the care process:

I think it would be really helpful to have a better understanding of creating an online learning environment that’s conducive to safety, to people feeling safe enough to ask questions, express values, and be clear about assumptions and be open. So just really helping to create that.

P2, interview, nursing student

Furthermore, students desired more opportunities to learn how to convey empathy in the virtual setting. One student described why they thought conveying empathy was important:

I might not be able to offer therapeutic touch for somebody who’s grieving, but I can show as much empathy as possible and possibly recommend them to additional resources. Because I feel like as people, it’s fundamentally important to validate how individuals feel and work toward improving their health. And I mean in a remote setting, that might take a few times, a few follow-ups just as in a physical setting, but you can make progress too.

P8, interview, nursing student

##### Care Management

Virtual care management requires critical thinking, organization, advocacy, adaptability, client education, assessment, documentation, and problem-solving, all important skills for students to develop. Students discussed the skills involved in organizing client care and serving as advocates to access resources in virtual care environments:

I think definitely, especially in this current environment of nursing that I’m in, you take on more responsibilities in terms of organizing and advocating for patients. So especially with remote care, I think that aspect of taking clerical roles on top of nursing while still providing adequate and best practice nursing care. I think that’s something that had I not had remote learning, I would’ve been less practiced in.

P1, interview, nursing student

Students also reflected on the skills that they needed to complete comprehensive client assessments in the virtual setting. They noted that as professionals, they needed to be skilled in asking the right questions and knowing when to dig a little deeper:

I think it made my assessments more comprehensive. Instead of just focusing on a person physically, there are a lot of questions that you can ask them that are objective that you don’t need to see a person face to face to ask, that you could quite literally do through a chat if you needed to. And I think it really honed in on, how do I put this, your ability to use resources to the best of your ability.

P8, interview, nursing student

Students expressed that adaptably was very important in the virtual setting. One student described how their educators’ adaptability provided benefits to their online learning during the early days of the pandemic and inspired them to mirror this behavior as one education student shared:

One of my professors, the methodologies course professor, also changed one of the learning tasks when we shifted online and shifted it from a research paper into a research presentation and had us partner up with someone in the class. It made it much more dialogic as opposed to written. I think this helped them both because they were transitioning their class online, which comes with a lot of logistical headaches and logistical work. It helped them cut down on their marking a little bit or change the way that they had to mark, and then it also allowed us to make sure we had someone we were regularly connecting with and dealing with the content with. So, that was nice. I would use those types of strategies moving forward.

P6, interview, education student

##### Technology Literacy

Technology literacy was noted as key to students’ ability to perform virtual care. Students indicated that they needed to learn and gain confidence in using various virtual care technology platforms. In addition, one student discussed how technology literacy was an asset in their work within health care and education:

So, with the remote care of patients, I found I’m much more comfortable with using the remote technology. So, it’s given me that comfort and it’s also given me the comfort of walking patients and their families through the remote technology and being able to access, walk them through how to access the chat functions or even able getting them to use their camera to show me specific things has been helpful.

P7, interview, nursing student

Students expressed the need to understand the legal and ethical implications of providing virtual care, including aspects of adequate documentation and privacy concerns:

Yeah, I think how to navigate some of the pitfalls and how to navigate some of the challenges to providing this type of care. I mean, I think there’s some issues around legalities and managing information that aren’t necessarily addressed clearly in your teaching and learning.

P2, interview, nursing student

#### Teaching Methods

Students discussed many teaching methods that their instructors used that helped them develop virtual care skills. These teaching methods were divided into 3 main categories: collaborative learning, experiential learning, and observational learning.

##### Collaborative Learning

Collaborative learning was found to contribute to students’ development of virtual care skills. Students discussed how class discussions and group work enriched their educational experience, emphasizing the positive impact of collaborative approaches:

I find I learn best through discussions and problem solving with other people. So I like it when we get projects to work on in smaller groups and then go back to a larger group online to present our work that we’ve worked together on in smaller groups.

P7, interview, nursing student

Underscoring the value of interdisciplinary collaboration in enhancing the skills related to providing virtual care, one student remarked on the benefits of participating in an interprofessional education sessions:

In previous terms we’ve done interviews with simulated patients where we collected health information, then worked with other students from other healthcare teams. I think that was really helpful in teaching. I think more of those interprofessional exercises would be really helpful going forward, just to kind of see how would you then work with another healthcare provider to provide care from different locations remotely. I think that would be helpful in continuing to do, and if not doing more.

P1, interview, nursing student

##### Experiential Learning

Experiential learning was noted by students to be important in developing virtual care skills. Specifically, students stated that engaging with virtual simulations, participating in role-play, and analyzing case-study scenarios significantly contributed to their understanding and acquisition of virtual care skills:

The learning opportunity for me was actually doing the role playing with my peers, but my teaching opportunity was also facilitating that role play online because during the pandemic, that was the only way that we were meeting with patients and clients out in the world. So it was that interaction online, learning that in the classroom to sort of set that skill set, get that skill set up to speed, so that when you’re actually interacting with that individual online in a virtual caring situation, there’s that transference of knowledge into what it is we were actually doing, what the students would be preparing for, for their clinical.

P5, interview, nursing student

Some students highlighted the benefit of a virtual practicum experience, where they had the opportunity to interact with actual clients in a virtual setting:

We had an assignment to interview someone with diabetes, we used videoconferencing to take a history from them, and received simulated medical records.

P85, survey, medical student

It would be lovely if it was a specific course on remote care, that would be fantastic. But if not a specific course, at least a few class days spent on perhaps assessing a patient remotely, like assess learning using either actual patients or acting patients and having the students have to learn how to or have to do a head-to-toe assessment or some sort of assessment on a patient who actually is remote just to get the practice of it.

P7, interview, nursing student

##### Observational Learning

Students discussed how they were able to learn virtual care skills by observing faculty and practicing professional work within a virtual setting:

Demonstration throughout clerkship has shown the many positives and negatives of virtual care.

P86, survey, medical student

In addition, an interview participant remarked on how they learned skills by observing others provide education in a virtual setting and thought about how they might use that in their own practice:

[Y]ou’ve had someone do a presentation and they just presented, their presentation skills were unique and interesting, and it’s something that you could learn to build on for yourself. I think you take away a lot from that, having that place to sandbox you’re learning and then bringing it into your formalized teaching or care practice that you’re doing remotely.

P2, interview, nursing student

This observational learning played a crucial role in student skill development.

#### Synthesis of Findings

In our quantitative survey analysis, we observed that most respondents (80/89, 90%) expressed some level of satisfaction with the online teaching and learning strategies that were used to help them gain virtual care skills. These results are congruent with the qualitative findings that indicated that while students reported primarily positive learning experiences, they also saw potential for improvement. For example, many students suggested incorporating more virtual care exposure in their programs of study to further develop virtual care skills.

In the quantitative survey, students reported varying levels of preparation , which was also corroborated by our qualitative data. Although some students had some exposure to virtual care curricula and practical experience, others reported minimal exposure and offered suggestions to better prepare future students to learn and work in virtual environments. All students emphasized the importance of virtual care skills in quickly changing caring environments.

In our quantitative survey, students identified various teaching methods that helped them to learn virtual care skills. These quantitative results aligned partially with our qualitative findings. We have provided a joint display to further describe the integration of these results in Table 3.

**Table 3:** Joint display of teaching method data (n=93).

Qualitative themes			Quantitative data, n (%)		Participant quote		Interpretation
**Collaborative learning**							
	Participants indicated that online discussion boards were used to develop their virtual care skills.	65 (70)		“We learn through reflection and that can easily be done virtually and on discussion boards.” [P22, survey, allied health]		Discussion boards were mentioned in the surveys and interviews as a way to collaborate with other students and faculty. Although it is a widely used teaching method, many students valued synchronous online discussions and group work as a way to collaborate and build connections.	
**Experiential learning**							
	Participants indicated that they were able to consult with clients to enhance their virtual care skills.	23 (25)		“Those are kind of things that I didn’t have any skills with when I graduated nursing, so that’s something I had to learn with experience and just on the job. So just giving the nursing students practice with these kinds of phone calls from either fake patients or families with scenarios of what’s going on, and have the nurse think about what advice they should give. And yeah, practicing doing virtual head to toe assessments, or not virtual, but yeah, online head to toe assessments of patients.” [P7, interview, nursing student]		Students expressed their desire for practical experience with virtual care while in their program; however, most students did not get the opportunity for this experience. Educational leaders should make an effort to provide virtual care practical experience to best prepare students for the challenges they will meet in the workplace.	
**Observational learning**							
	Participants indicated that they had had the opportunity to watch a demonstration of virtual care.	19 (20)		“Most of my learning has been from watching physicians do virtual care.” [P80, survey, medical student]		Students benefited from seeing practicing professionals demonstrate proper virtual care; however, only a small percentage were provided that opportunity. This gap can be addressed by offering virtual care practicums or having faculty demonstrate effective virtual care.	
**Divergence (no theme)**							
	Participants indicated that online modules were used to further their learning of virtual care skills.	77 (83)		—^a^		Although online modules can be helpful and are widely used to organize course content, students value the education more when there is an effort made to ensure learning has collaborative, experiential, and observational elements.	
	Participants indicated that reflection was used to help develop virtual care skills.	66 (71)		—		Although most survey respondents identified reflection on learning as a teaching method that helped them learn virtual care skills, this is not something that was explicitly discussed in the interviews and qualitative survey responses. This could be because many of the teaching methods proposed inherently use reflection (eg, simulation, discussion boards, group discussion).	

^a^Not available.

## Discussion

### Principal Findings

In this mixed methods study, our primary objective was to explore the experiences and perspectives of caring profession students concerning online learning opportunities aimed at enhancing their virtual care skills and competencies. We sought to uncover both the facilitators and barriers to creating and engaging in such online learning opportunities. We conducted surveys and interviews with caring profession students from education, medicine, nursing and allied health disciplines across a midsized research-intensive educational institution located in western Canada. While no statistically significant differences were noted in satisfaction scores or preparedness scores based on gender, age, faculty, or experience with online teaching and learning technologies, the survey combined with the interview data revealed a number of interesting findings.

Students identified several barriers to effective online learning in the context of developing virtual care skills for future practice. These difficulties comprised challenges with technology, lack of accessibility, and lack of personal connection. Providing virtual care has undoubtedly revolutionized caring professions while also raising challenges that can act as barriers to effective virtual care delivery. Challenges with technology can be a significant hurdle, especially in cases where not all students or clients have access to reliable internet connections or the necessary devices for virtual care [19,20]. Although not explicitly mentioned by student participants, these challenges can disproportionately affect vulnerable populations, hindering their ability to provide and receive adequate care [20]. Moreover, technical glitches and software issues can disrupt virtual appointments, causing frustration and potentially compromising client safety. The lack of accessibility to virtual care may also be problematic. Some students and clients may require special accommodations, such as sign-language interpreters or Braille interfaces, which may not be readily available in virtual settings. This lack of inclusivity may lead to suboptimal learning and care for those with disabilities or language barriers. Finally, the limitations on personal connection in virtual care can potentially impede the caring relationship [21]. The physical distance can make it challenging for caring professionals to establish rapport, interpret nonverbal cues, and provide empathetic care. Students and clients may also feel isolated or detached from their educators or care providers, which can affect the quality of their learning or caring experiences. While virtual care offers many advantages, it is imperative to consider and address these barriers to ensure equitable, accessible, and client-centered care for all. Educators, students, administrators, policy makers, and caring professionals must work together to bridge these gaps and harness technology to optimize its full potential in virtual care learning and delivery. Ultimately, a more concerted effort can lead to a future where virtual care is maximized to benefit both students and their recipients of care.

Facilitating the development of virtual care skills in caring profession students requires a multifaceted approach that includes reliable technology infrastructures, expert faculty, a comprehensive remote care curriculum, and ample remote care exposure. Postsecondary educational institutions need to provide the technologies required for robust online learning environments. Expert faculty can play a pivotal role in guiding students toward mastering virtual care skills. Drawing from their wealth of knowledge and clinical expertise, experienced educators can provide valuable insights, feedback, and real-world scenarios to nurture students’ empathy and communication, as well as help students understand the nuances of delivering compassionate care in remote settings. Well-designed remote care curricula are needed to not only cover the technical aspects of virtual care but also emphasize the importance of empathy, active listening, and cultural sensitivity. Such curricula should provide case studies, simulations, and role-playing exercises to allow students to practice and refine their virtual care skills in controlled environments [22]. Caring professional students must have ample opportunities for remote care exposure. This may include clinical rotations, internships, or practical experiences where students interact with patients virtually under expert faculty guidance. These hands-on experiences allow students to apply their theoretical knowledge, develop their virtual care skills, and gain confidence in providing compassionate care through digital platforms [23,24]. When combined, these elements can effectively work together to prepare future caring professionals to deliver high-quality, client-centered care irrespective of the mode of communication.

In a virtual environment, mastering caring skills becomes even more crucial, and several key factors should guide the learning and demonstrating of these skills in practice [25]. Participants in this study identified five principles of virtual care: (1) client centered, (2) connection, (3) inclusive and accessible, (4) engagement, and (5) flexibility. Prioritizing the needs and preferences of the student or client is paramount. Virtual care should be tailored to the individual’s unique circumstances and requirements to ensure that their concerns, values, and goals remain centered [26]. Caring professionals must actively engage with clients to foster trust and rapport through effective communication and active listening. Showing empathy and a caring demeanor can help strengthen this connection, and ensuring that virtual care is accessible to all is essential [27]. This may include accommodating individuals with disabilities, providing language translation services, and addressing cultural sensitivities to ensure that every student and client feels included and valued. While maintaining engagement in a virtual environment can be challenging due to distractions and technology-related issues, caring professionals can use strategies to keep clients actively involved in their care, promoting adherence to care plans and shared decision-making. Flexibility is also essential in adapting to the unique circumstances of each student and client. Virtual care providers should be ready to adjust their approach, timing, and content to meet the evolving needs of their clients, allowing for personalized care plans [28]. Client-centered, connection-focused, inclusive, accessible, engagement-driven, and flexible virtual care practices are critical components for effectively demonstrating caring skills. These factors can ensure that caring professionals can deliver compassionate, high-quality care in the digital realm, maintaining the dignity and well-being of their clients while adapting to the ever-evolving landscape of virtual care delivery.

Although student participants provided thoughtful responses, their limited exposure to virtual care may have hindered their ability to give informed perspectives. When considering these perspectives, it is important to reflect on the guidelines offered by professional organizations and the government to identify areas that may not have been captured in student interviews. The College of Physicians and Surgeons of Alberta has published principles for virtual care including providing high-quality care, upholding ethical and legal standards, ensuring the appropriateness of the use of virtual care as well as patient privacy and confidentiality [29]. The government of Canada’s Virtual Care Policy Framework has four policy pillars, including (1) community and patient-centered approaches; (2) equitable access to care; (3) provider incentive and payment; and (4) appropriateness and quality of care, provider change management, and licensure [30]. In addition, the American Telemedicine Association has published principles for virtual health providers, including upholding legal and ethical standards, upholding the standard of care regardless of care modality, maintaining appropriate licensure, mitigating threats to confidentiality and patient safety, and avoiding misleading advertisements [31]. Although there are organizations that support online education practices, they do not speak to the concept of virtual care through education, indicating a potential gap in guidance for this caring profession. Although student participants offered valuable insights into more individual-level concerns, they may not have been exposed to practical system concerns such as legal and ethical standards, appropriateness of care modality, equity, cybersecurity, and provider payment structures. These may also be considered as important content areas when developing a curriculum to prepare students for practice.

To deliver effective virtual care, students in caring professions need to cultivate a combination of relational skills, care management skills, and technology literacy. These skills are indispensable in ensuring high-quality, client-centered care in virtual care environments. Building strong relationships is important in providing virtual care, and caring professionals must excel in active listening, empathy, and effective communication to establish trust, understand client’s needs, and provide emotional support remotely [32-36]. These skills enable caring professionals to connect with clients on a personal level and deliver compassionate, comforting care. In virtual settings, effective care management can involve comprehensive assessment, treatment planning, and monitoring. Caring profession students need to develop skills in remote assessment, risk evaluation, and care coordination to become adept at developing individualized care plans, setting goals, and tracking progress while addressing any challenges that arise in the virtual care environment. A strong foundation in technology literacy is essential for navigating the digital tools and platforms used in virtual care [37-39]. Caring professionals should be proficient in using virtual connection software, electronic records, and other digital tools to facilitate consultations, securely access client data, and ensure compliance with privacy regulations. Being technology savvy not only improves efficiency but also enhances the overall client experience by minimizing technical disruptions during virtual appointments. The development of relational skills, care management skills, and technology literacy is crucial for students in the caring profession to excel in providing virtual care, establish meaningful connections with clients, manage care effectively in remote settings, and leverage technology to benefit the clients they care for.

In this study, students identified three impactful educational approaches that significantly contribute to the development of virtual care skills: (1) collaborative learning, (2) experiential learning, and (3) observational learning. Collaborative learning, whether through group discussions, case studies, or collaborative projects, offers students a platform to exchange ideas, insights, and feedback. Engaging in discussions with peers exposes students to diverse client scenarios, fosters empathy, and facilitates a deeper understanding of different perspectives on care. It also encourages students to collectively engage in problem-solving, stimulating the creation of innovative strategies for delivering compassionate care in virtual settings. Learning by doing is another way to master virtual care skills. Practical experiences, such as virtual clinical simulations or telehealth practice sessions, enable students to apply their theoretical knowledge in real-world scenarios [22]. Through these hands-on experiences, students can practice active listening, effective communication, and empathetic responses while addressing the needs of virtual patients. Experiential learning builds confidence and hones the skills necessary for effective virtual care delivery [39]. Students can gain insights into best practices for virtual care by observing skilled practitioners during virtual consultations. This observational learning allows them to identify effective communication techniques and develop empathetic responses and strategies for building patient rapport, which they can then integrate into their own practice. Finally, a combination of collaborative, experiential, and observational learning may provide a well-rounded education that equips students with the virtual care skills needed in contemporary virtual environments. These approaches can encourage active engagement, practical application, and exposure to best practices, ultimately preparing students to deliver compassionate and effective care in virtual environments.

### Strengths and Limitations

This study used a sequential explanatory mixed methods design, offering a robust exploration of virtual care skill development within a specific educational institution. The inclusion of participant learners from diverse caring professions provided a rich array of perspectives, enriching the comprehensiveness of the study. Using surveys and interviews, the study integrated both quantitative and qualitative data, allowing for a deeper understanding of students’ experiences and perspectives in developing competencies related to virtual care. However, the limitations of our study suggest the need for some caution when interpreting our findings. The study’s focus on a single institution could potentially constrain the generalizability of our findings to broader settings, as the findings may be specific to that particular institution. In addition, the participant pool being drawn from a single institution might introduce a lack of diversity in perspectives, impacting the external validity and transferability of our findings to a more varied population.

Furthermore, it is possible student respondents may have interpreted the terms in the survey regarding virtual care differently than the research team intended, as the terms were not explicitly defined. Most survey respondents (89/93, 96%) reported teaching methods that were used to enhance their virtual care skills. Given the lack of a formal virtual care curriculum discussed by the students in interviews, our qualitative sample might be biased. Despite these limitations, our study serves as a foundational exploration of virtual care skill development, encourages further research in this area, and promises potential advancements in understanding and improving the delivery of virtual care in educational settings.

### Conclusions

Caring professionals require specialized knowledge and skills to deliver high-quality, effective virtual care; however, little is known about what is needed to teach students in this skill set. Our study highlights the barriers, facilitators, and principles of learning virtual care skills while also identifying pertinent skills and impactful teaching methods. This study contributes to the growing body of educational research regarding virtual care skills by providing insights into student perspectives and offering suggestions for optimizing teaching and learning strategies within caring professions’ educational programs.

## Abbreviations

SRQR: Standards for Reporting Qualitative Research
